# Interfacial nanoarchitectonics for ZIF-8 membranes with enhanced gas separation

**DOI:** 10.3762/bjnano.13.26

**Published:** 2022-03-22

**Authors:** Season S Chen, Zhen-Jie Yang, Chia-Hao Chang, Hoong-Uei Koh, Sameerah I Al-Saeedi, Kuo-Lun Tung, Kevin C-W Wu

**Affiliations:** 1 School of Energy and Environment, City University of Hong Kong, Kowloon, Hong Kong SAR, 999077, China; 2 Department of Chemical Engineering, National Taiwan University, No. 1, Sec. 4, Roosevelt Road, Taipei 10617, Taiwan; 3 Department of Chemistry, College of Science, Princess Nourah bint Abdulrahman University P.O.Box 84428. Riyadh 116711, Saudi Arabia

**Keywords:** defect-free, gas separation, interfacial synthesis, metal-organic frameworks, ZIF membranes

## Abstract

Metal-organic framework (MOF) membranes are potentially useful in gas separation applications. Conventional methods of MOF membrane preparation require multiple steps and high-pressure conditions. In this study, a reliable one-step interfacial synthesis method under atmospheric pressure has been developed to prepare zeolitic imidazolate framework-8 (ZIF-8) membranes supported on porous α-Al_2_O_3_ disks. To obtain optimal ZIF-8 membranes, three reaction parameters were investigated, namely, reaction temperature, reaction time, and concentration of the organic linker (i.e., 2-methylimidazole). The growth of ZIF-8 membranes under various parameters was evaluated by field-emission scanning electron microscopy, and the optimal synthesis conditions were determined (i.e., 80 °C for 12 h in 50 mM of 2-methylimidazole). The as-synthesized ZIF-8 membranes were then applied to CO_2_/N_2_ gas separation, which exhibited a maximum separation factor of 5.49 and CO_2_ gas permeance of 0.47 × 10^−7^ mol·m^−2^·s^−1^·Pa^−1^.

## Introduction

Carbon dioxide (CO_2_) is one of the major greenhouse gases emitted through human activities contributing to global climate change. According to United States Environmental Protection Agency (US EPA), CO_2_ emission has increased by about 90% since 1970, and the global mean CO_2_ level is over 410 ppm in 2020 [1]. More than 160 nations signed the Paris Agreement in 2016, committing to combat global warming by cutting CO_2_ emissions by 49% by 2030 [2–3]. To meet this goal, CO_2_ capture and storage (CCS) from flue gas in power plants presents a promising route [4]. There are mainly three types of CO_2_ recovery systems, namely, pre-combustion, post-combustion, and oxyfuel combustion. After the recovery of CO_2_, separation of CO_2_ from N_2_ (i.e., the main gas in power plant flue gas) or CH_4_ (i.e., the main gas in natural gas) with high efficiency is needed.

Membrane-based separation offers a great potential for online CO_2_ sequestration in view of its high energy efficiency, small carbon footprint, and competitive cost compared to traditional separation processes, such as distillation and adsorption [5]. The current membrane separation market in chemical engineering is dominated by polymeric membranes because of their low cost, ease of production, and mechanical flexibility [6–9]. However, applications of polymeric membranes in the separation processes are limited by their short lifetime and inferior chemical and thermal stability. Zeolite membranes, in contrast, are attractive for separation processes in harsh chemical environments and at high temperatures, owing to their high chemical and thermal stability. Additionally, these inorganic membranes possess relatively uniform pore structures and give rise to specific molecule-sized pores, which yield a high separation factor in the separation processes [9–12]. Wider application of zeolite membranes in separation is limited by the narrow pore-size range (0.2–2 nm) and the difficult chemical modification.

Metal-organic frameworks (MOFs) are a novel class of porous crystalline materials formed by interconnecting organic linkers and metal ions. They possess a high accessible internal surface area (typically 500–7000 m^2^·g^−1^) [13–14]. In comparison to zeolite membranes, the synthesis conditions of MOF membranes are less energy intensive. Unlike zeolite membranes, MOF membranes do not need structure-directing agents in the fabrication process. In addition, the pore sizes and surface functionalities of MOF membranes can be chemically modified, which allows for a rational design to separate various sizes of molecules [13–17]. Given these structural properties, MOFs are widely applied to gas storage [18], gas/liquid separation [18–20], energy storage [21–23], sensing [24], catalysis [25], electrochemistry [26], and bio-related fields [27].

Zeolitic imidazolate frameworks (ZIFs), a subclass of MOFs, comprise imidazoles (organic linkers) and zinc or cobalt ions (metal ions). They are considered as promising nanostructured materials in technologies related to energy and environmental science [2,28–30]. ZIFs are of particular interest for gas separation due to their additional framework flexibility imposed by rotation of the organic linkers [19,31], besides their remarkable thermal and chemical stability [32]. The linker rotation effect can mediate guest molecule diffusion efficiently since larger molecules encounter a larger hindrance by the rotating linkers. ZIF-8 exhibits this representative linker rotation effect through the methyl group of 2-methylimidazole. In addition to providing selectivity, ZIF-8 also possesses a high volume of cavities in which the open framework structure provides a passing lane for small molecules while large molecules are trapped [31]. The crystallographic pore aperture of ZIF-8 is 3.4 Å, which is deemed ideal for CO_2_ separation since the kinetic diameters of CO_2_, N_2_, and CH_4_ are 3.3 Å, 3.6 Å, and 3.8 Å, respectively [33–34].

The performance of MOF-based membrane separation highly depends on the microstructure and crystal structure of the selective layer, while the intercrystalline defect formation in MOFs can have either positive or negative effects on the separation performance. Point defects and extended defects may increase the number of adsorption sites in MOFs [35], while missing linkers may provide low-resistance diffusion pathways by increasing the porosity of the MOF [36]. However, the presence of defects can hamper the structural stability of MOFs [35]. Therefore, characterization of defects in MOF-based membranes and the correlation to the preparation methods with membrane properties are critical in MOF film development. Among the various synthesis schemes, the interfacial synthesis method confines the coordination of the MOF to the solvent interface, which ensures good control over MOF nucleation and growth processes [37–38]. Consequently, it is a promising approach to synthesize a defect-free MOF film. In comparison, the counter-diffusion method usually separates the metal ion and ligand solutions by a porous substrate, and crystallization occurs within the substrate. Since the diffusion rates of metal ions and ligands are usually different due to different interactions with the substrate, the resultant membranes are likely to contain defects.

In order to understand (a) the relationship between synthesis method and intercrystalline structure, (b) the relationship between ZIF-8 crystal structural in the film and the associated gas separation performance, and (c) the effect of α-Al_2_O_3_ support on membrane separation performance, free-standing ZIF-8 films were synthesized via an interfacial method, while an interfacial method and a counter-diffusion method were adopted to synthesize α-Al_2_O_3_-supported ZIF-8 membranes for CO_2_/N_2_ gas separation.

## Materials and Methods

### Chemicals

Zinc nitrate hexahydrate, 2-methylimidazole (2-MIM), sodium formate of reagent grade, methanol and 1-octanol of analysis grade were purchased from Sigma-Aldrich. Deionized water produced with a Merck Millipore system was used. The α-Al_2_O_3_ disks (30 mm in diameter and 1 mm in thickness) were purchased from Fraunhofer IKTS.

### Synthesis of ZIF-8 free-standing films

Zinc nitrate hexahydrate (1 mmol) was first dissolved in deionized water (10 mL) in a vial (20 mL). 2-Methylimidazole (0.5 mmol) and sodium formate (0.5 mmol) were added to 1-octanol (10 mL). To completely dissolve 2-methylimidazole, the 1-octanol solution was stirred for at least 30 min. Then, the 2-methylimidazole/1-octanol solution was added dropwise to the zinc nitrate solution. The mixture was kept at 80 °C in an oil bath for 12 h to form a ZIF-8 free-standing thin film on the liquid–liquid interface. After the reaction, fragments of the ZIF-8 thin film were dispersed in methanol to remove solvents and unreacted reactants. Then, these fragments were collected by centrifugation at 25,000 rpm for 10 min. This washing process was repeated for three times. The resultant products were placed in a lyophilizer for at least 12 hours for drying.

### Synthesis of ZIF-8 membranes via an interfacial synthesis method

A porous α-Al_2_O_3_ disk was placed in a lyophilizer for at least 6 h to remove adsorbed water or solvents. 2-Methylimidazole and sodium formate (0.5 mmol) were added to 1-octanol (35 mL), and zinc nitrate hexahydrate (5 mmol) was dissolved in deionized water (50 mL). Different concentrations (12.5–400 mM) of the 2-methylimidazole/1-octanol solution were prepared to optimize the ZIF-8 membrane structure. Since 1-octanol is immiscible with water, ZIF-8 formation can be confined to the interface between water and the organic solvent. Prior to ZIF-8 crystal growth, the pretreated α-Al_2_O_3_ disk was immersed into the zinc nitrate aqueous solution (50 mL) in an Erlenmeyer flask. The flask was connected to an aspirator to provide a vacuum to remove the air inside the pores of the disk. Then, the disk was kept in the aqueous solution for another 30 min until the porous support was fully filled with zinc nitrate. The α-Al_2_O_3_ disk was taken out of the solution, and the excess solution on the support surface was removed with a rubber wiper. The disk was gently immersed vertically into the preheated 2-methylimidazole/1-octanol solution (35 mL) at different temperatures (40–120 °C) in a beaker placed in an oil bath for the growth of ZIF-8 membranes. After the desired reaction time (3–15 h), the mixture was cooled down to room temperature in air. The cooled ZIF-8 membrane was immersed into methanol (50 mL) for 1 h to remove most of the unreacted reactants and solvents. Then, the ZIF-8 membrane was transferred into fresh methanol (50 mL) for 11 h for further purification. The beaker covered with parafilm was then placed in an oven at 45 °C for at least 12 h to dry the ZIF-8 membrane in saturated methanol vapor environment. Finally, the dried membrane was stored in a lyophilizer.

### Synthesis of ZIF-8 membranes via a counter-diffusion synthesis method

The porous α-Al_2_O_3_ disk was pretreated by the method described in the previous section. 2-Methylimidazole (1.75 mmol) and sodium formate (1.75 mmol) were added to deionized water (35 mL), and zinc nitrate hexahydrate (5 mmol) was dissolved in deionized water (50 mL). ZIF-8 membranes were synthesized according to the procedures described in the previous section with a reaction temperature of 80 °C and a reaction time of 12 h.

### Characterizations

Pore size and porosity of α-Al_2_O_3_ disks were determined with a mercury porosimeter (AutoPore^®^ IV 9520). Wide-angle patterns of powder X-ray diffraction were measured on a Rigaku Ultima IV with Cu Kα radiation (λ = 1.5418 Å) to check the crystallinity of the synthesized ZIF-8 thin films. The morphology of the supports, the free-standing thin films, and the supported membranes was observed by FE-SEM (Nova^TM^ NanoSEM 230). The elemental mappings of the films were obtained by energy-dispersive spectroscopy (EDS) connected with FE-SEM (Nova^TM^ NanoSEM 230).

### Single gas permeation

The synthesized ZIF-8 membrane was sealed in a stainless permeation module with two silicone O-rings on each side of the disc. Before individual permeation measurements of N_2_ and CO_2_, the membrane was swept by the target gas for at least 10 min to remove other gaseous species from the measurement system. Then, the side of the ZIF-8 selective layer was fed with pressurized target gas to provide a driving force for the gas to permeate through the membrane. The other side of the membrane was connected to a soap-film flow meter downstream to measure the gas permeation volume of the target gas. The pressure drop between feed side and permeation side was measured by a pressure meter (Bronkhorst EL-PRESS) and was kept at 20 psi. The temperature of the system was kept at room temperature (25 °C). The permeance *P*_i_ (mol·m^−2^·s^−1^·Pa^−1^) of the permeation gas was determined using the following equation:


[1] $$P_{\text{i}}=N_{\text{i}}/\Delta P_{\text{i}}A,$$

where *N*_i_ (mol·s^−1^) is the permeation molar flow rate of component gas i, Δ*P*_i_ (Pa) is the trans-membrane pressure drop of gas i, and *A* (m^2^) is the effective membrane area.

The ideal separation factor α of gas species i with respect to gas species j was defined by the following equation:


[2] $$\alpha_{\text{i/j}}=P_{\text{i}}/P_{\text{j}},$$

where *P*_i_ and *P*_j_ are the permeances of gas component i and j, respectively. The permeation flow volume of the target gas was recorded every 30 min during the measurement until the calculated permeation reached steady state.

## Results and Discussion

### Characterizations of porous α-Al_2_O_3_ disks and free-standing ZIF-8 thin films

Porous α-Al_2_O_3_ disks were chosen as ZIF-8 membrane supports, because they tolerate relatively high temperatures up to 2000 °C and harsh chemical environments (e.g., strong acids or bases or organic solvents) compared to polymer substrates. The particle size of α-Al_2_O_3_ on dense top layers of these disks is around 200 nm (information from Fraunhofer IKTS, Germany). The porosity of the disks was measured as 41.58% by mercury porosimeter measurements. The XRD patterns and SEM images of the α-Al_2_O_3_ disks are given in Supporting Information File 1, Figure S1.

**Figure 1:**
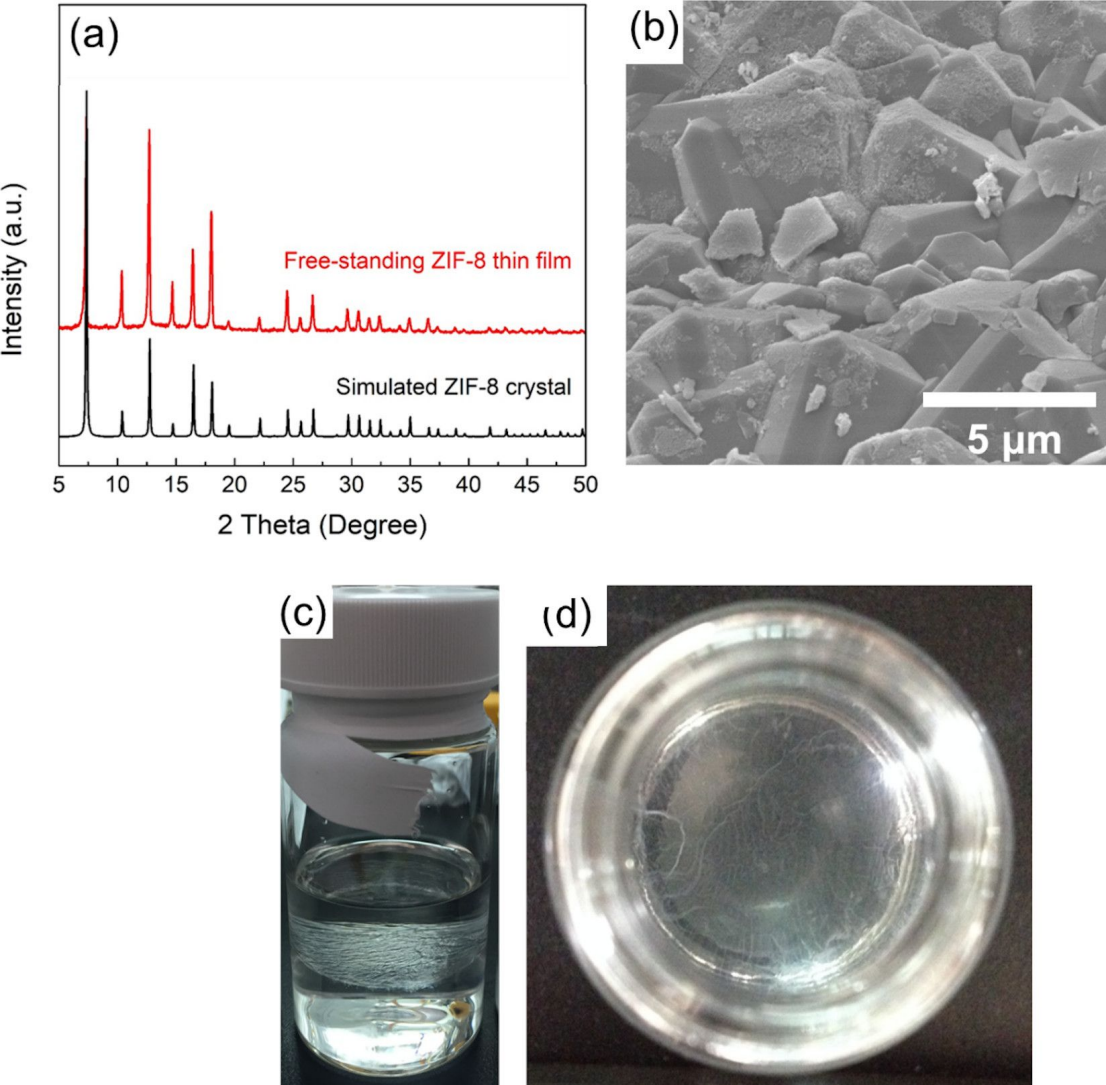
(a) XRD patterns, (b) FE-SEM image, (c) side view, and (d) top view of the free-standing ZIF-8 thin films.

Prior to heterogeneous nucleation of ZIF-8 crystals on the porous α-Al_2_O_3_ disks, free-standing ZIF-8 thin films were fabricated to verify the feasibility of continuous ZIF-8 crystal growth via an interfacial synthesis method. Figure 1a shows that the XRD patterns of the as-synthesized free-standing ZIF-8 thin film was identical to that of a ZIF-8 crystal simulated with data from Crystallography Open Database (Mercury, version 3.6), indicating the success of the synthesis of ZIF-8 crystals by interfacial synthesis. The SEM image shows that the ZIF-8 crystals have a grain size of approximately 5 μm (Figure 1b). A layer of white ZIF-8 crystals was clearly formed at the interface between aqueous and organic phases (Figure 1c,d).

### Characterizations of ZIF-8 membranes

**Figure 2:**
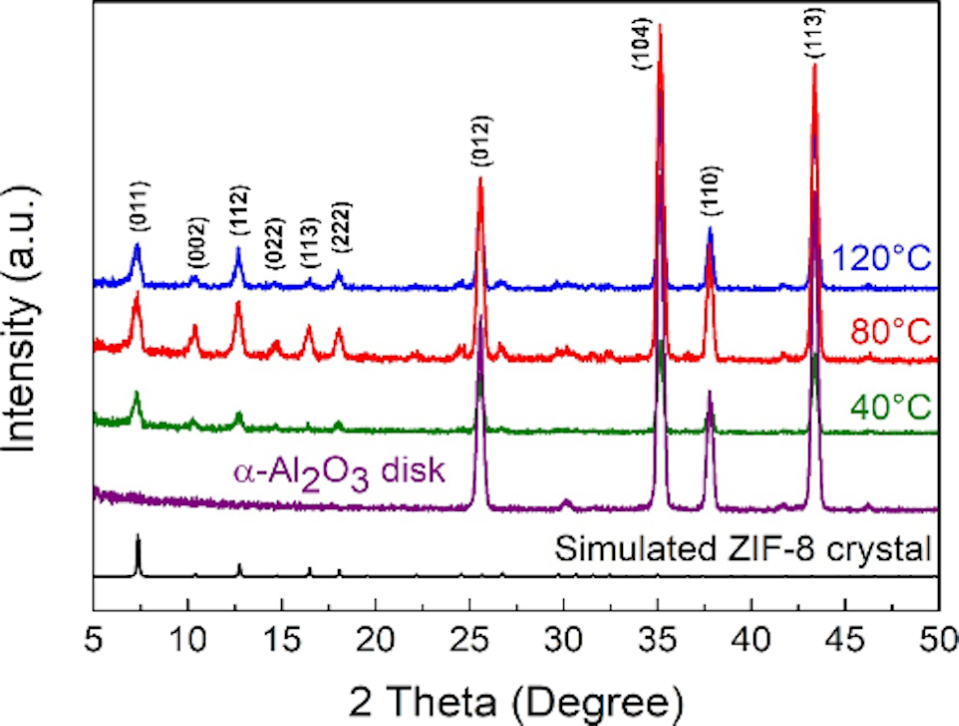
XRD patterns of simulated ZIF-8 crystal, porous α-Al_2_O_3_ disk, and ZIF-8 membranes fabricated at 40, 80, and 120 °C.

The XRD patterns of ZIF-8 membranes synthesized at different reaction temperatures (40, 80, and 120 °C) confirmed that the ZIF-8 membranes were composed of ZIF-8 crystals and α-Al_2_O_3_ disks (Figure 2). These results indicated that ZIF-8 crystals were successfully synthesized on porous α-Al_2_O_3_ disks, and the crystalline structure of α-Al_2_O_3_ on the support did not change. The intensity ratio of the strongest XRD peaks between ZIF-8 crystals ((011) at ca. 7.3°) and α-Al_2_O_3_ crystals ((104) at ca. 35.1°) was adopted as an indicator of ZIF-8 crystallinity, since the crystallinity of all commercial α-Al_2_O_3_ disks was consistent. Therefore, a higher intensity ratio indicated a higher crystallinity of ZIF-8. As the synthesis temperature increased from 40 to 120 °C, the intensity ratio increased from 23.44 to 45.90% (Table 1), suggesting a higher crystallinity of ZIF-8 synthesized at higher reaction temperatures.

**Table 1:** Intensity ratios of ZIF-8 membranes synthesized at different reaction temperatures.

	Reaction temperature (°C)		
	
	40	80	120

Intensity ratio^a^ (%)	23.44	30.33	45.90

^a^Intensity ratio = *I*_001_/*I*_104_ × 100%, where *I*_001_ (a.u.) represents the (001) reflection of ZIF-8 crystals, and *I*_104_ (a.u.) represents the (104) reflection of the porous α-Al_2_O_3_ disk.

**Figure 3:**
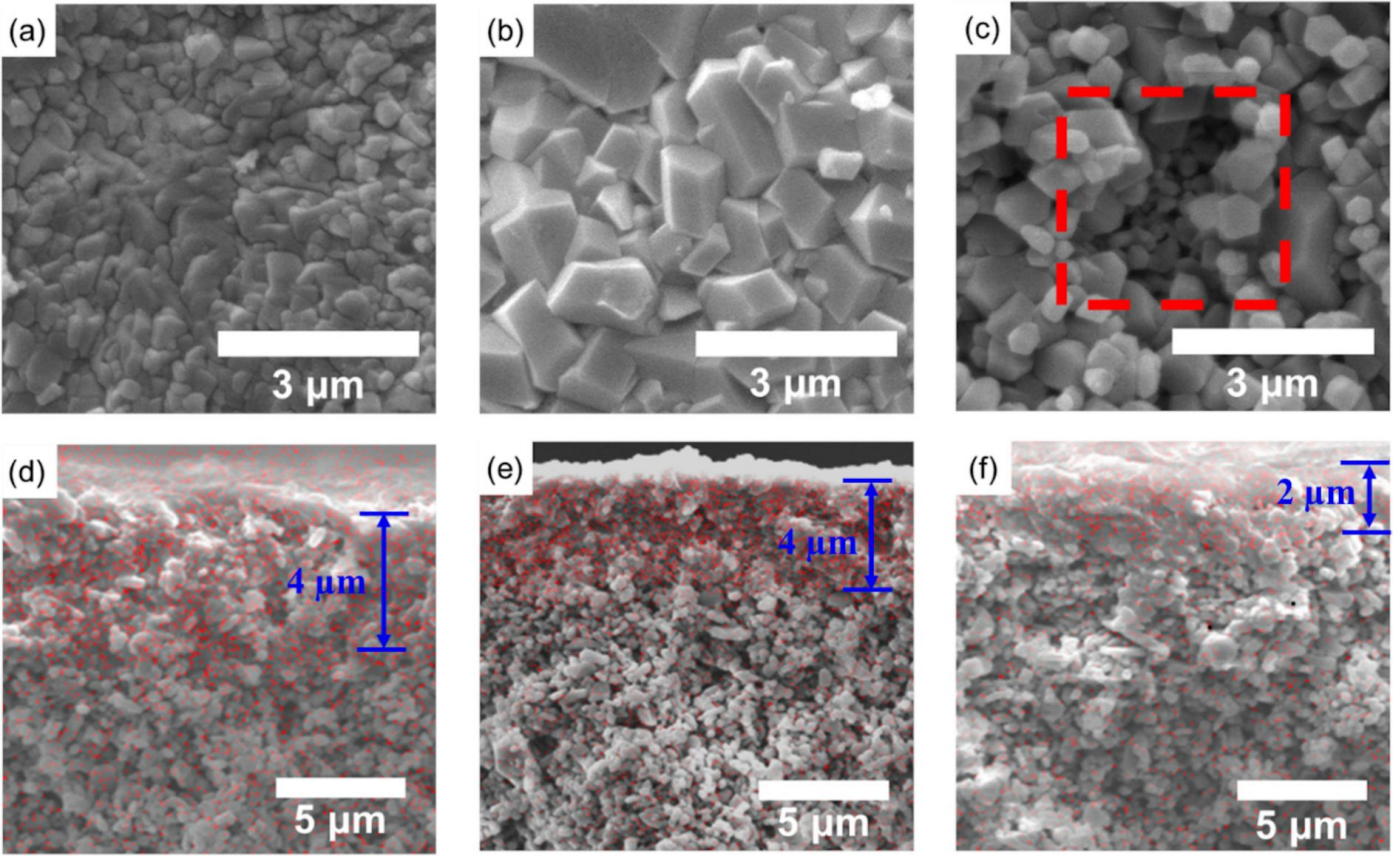
Top-view SEM images of ZIF-8 membranes fabricated at (a) 40 °C, (b) 80 °C, and (c) 120 °C, after 12 h. Cross-sectional EDS mappings of ZIF-8 membranes fabricated at (d) 40 °C, (e) 80 °C, and (f) 120 °C, after 12 h (red represents Zn).

Grain size of ZIF-8 crystals and membrane thickness were obtained from the FE-SEM images. The average grain size of ZIF-8 crystals synthesized at 80 °C was around 1.3 μm, which was about 2.6 times larger than the crystals synthesized at 40 °C (ca. 0.5 μm) (Figure 3a,b). Clear sodalite topology was observed in the ZIF-8 crystals synthesized at 80 and 120 °C (Figure 3b,c), which was in line with the higher crystallinity at higher synthesis temperatures indicated in Table 1. When the synthesis temperature increased to 120 °C, the particle size of ZIF-8 crystals was smaller than 500 nm (Figure 3c). Although a higher reaction temperature led to a higher crystallinity structure of ZIF-8, the surface of the membrane synthesized at 120 °C displayed exposed α-Al_2_O_3_ particles about 3 μm in length (Figure 3c). The uneven formation of ZIF-8 crystals on the membrane surface can be ascribed to the vapor bubbles from boiling water at 120 °C that disturbed the interface of aqueous and organic phases where ZIF-8 was synthesized. In addition, it can be seen from the cross-sectional images that the thickness of the ZIF-8 membrane was approximately 4 μm when the reaction temperature was 40 and 80 °C (Figure 3d,e), while the membrane thickness decreased to 2 μm when the reaction temperature increased to 120 °C (Figure 3f). The thinner membrane thickness can also be attributed to the disturbed liquid–liquid interface during synthesis.

**Figure 4:**
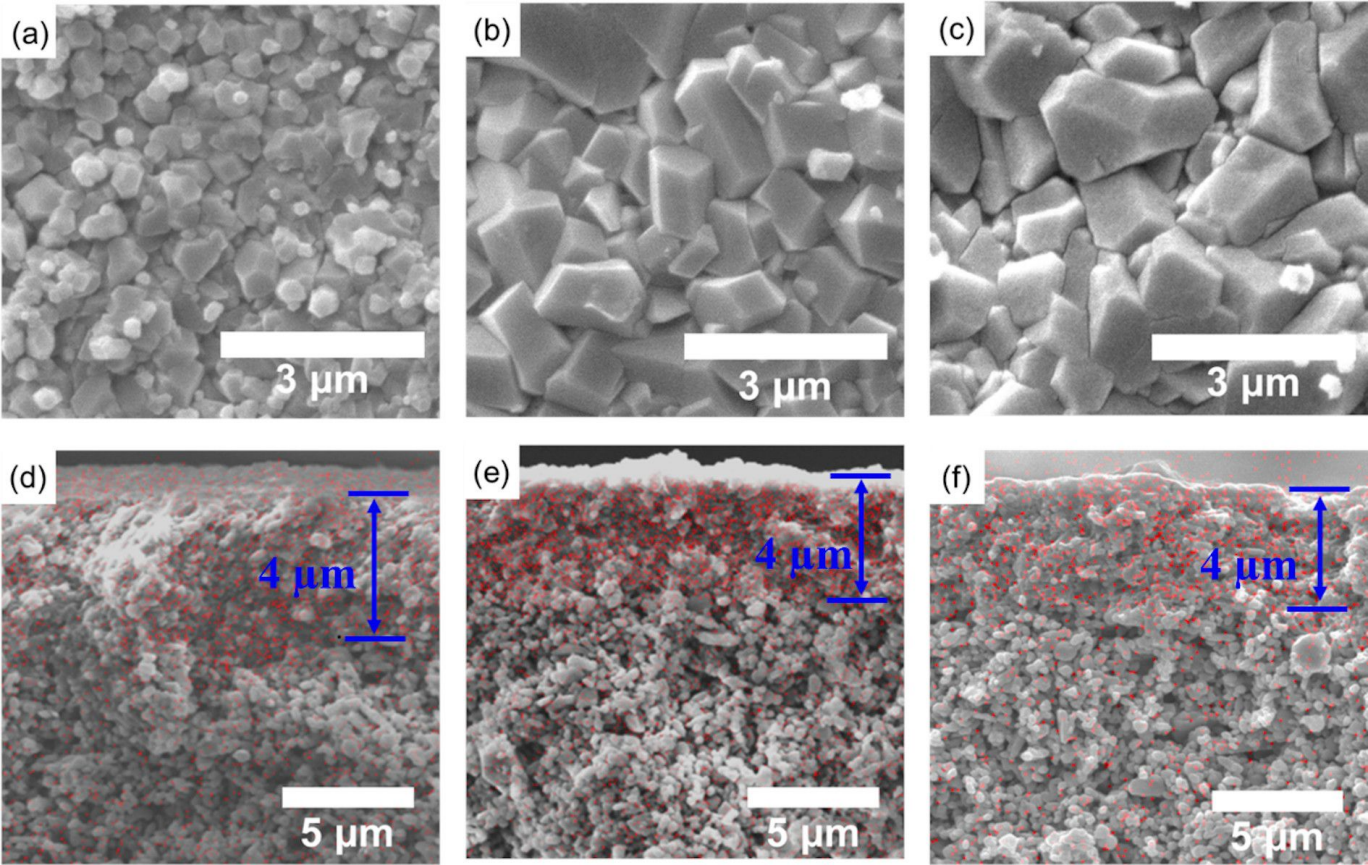
Top-view SEM images of ZIF-8 membranes obtained after (a) 3 h, (b) 12 h, and (c) 15 h at 80 °C. Cross-sectional EDS mappings of ZIF-8 membranes obtained after (d) 3 h, (e) 12 h, and (f) 15 h at 80 °C.

The growth of ZIF-8 crystals was also investigated regarding different reaction times. The SEM images showed that the crystal size increased from 0.6 to 1.1 μm when the reaction time increased from 3 to 12 h (Figure 4a,b), while the crystal size did not grow when the reaction time was further extended to 15 h. More interestingly, the thickness of ZIF-8 membranes was 4 μm regardless of the reaction time (Figure 4d–f). The formation of a micrometer-thick membrane is consistent with the results of interfacial syntheses of other MOF membranes [39–40].

**Figure 5:**
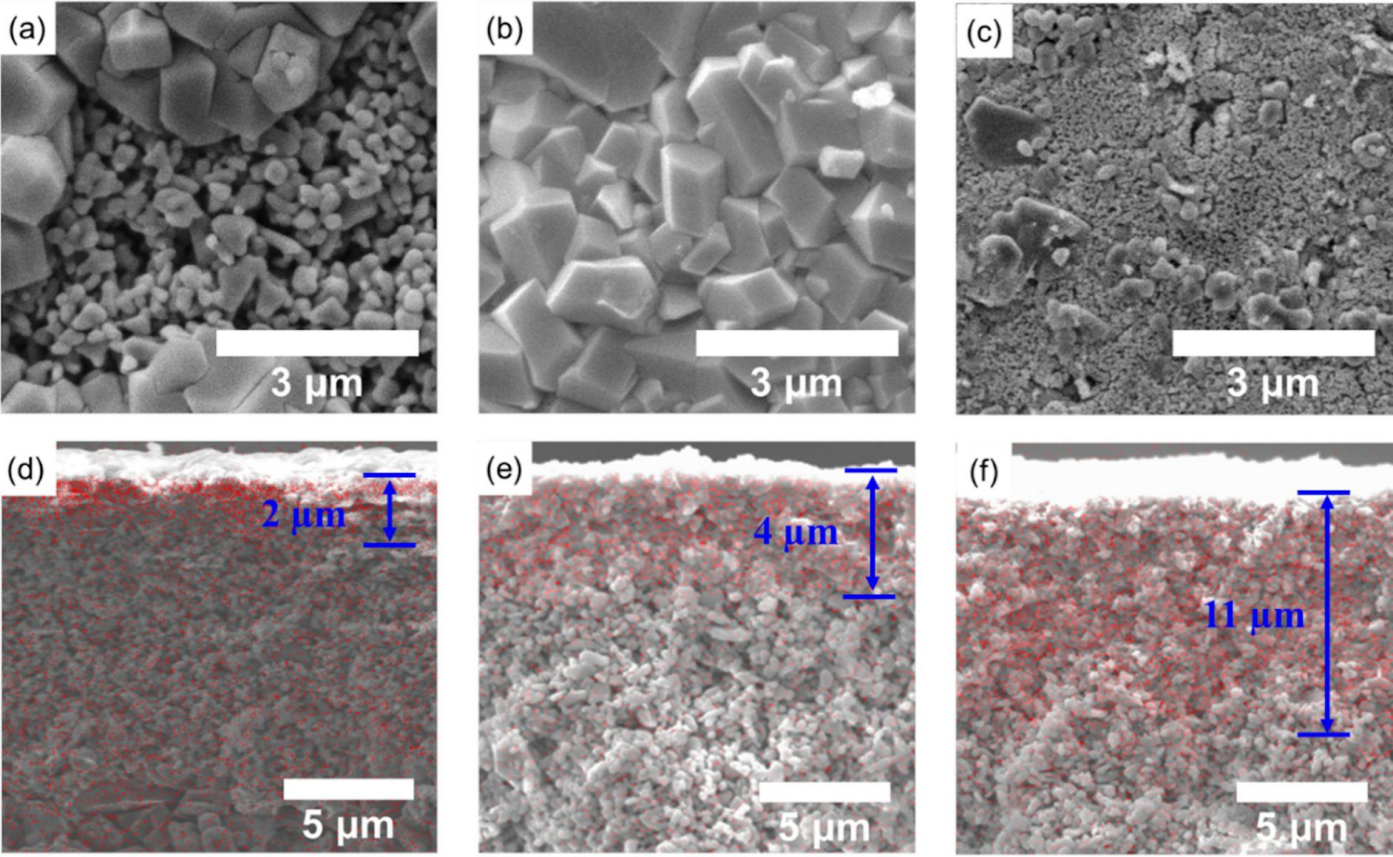
Top-view SEM images of ZIF-8 membranes fabricated with 2-methylimidazole/1-octanol solution concentration of (a) 25 mM, (b) 50 mM, and (c) 400 mM after 12 h at 80 °C. Cross-sectional EDS mappings of ZIF-8 membranes fabricated with 2-methylimidazole/1-octanol solution concentration of (a) 25 mM, (b) 50 mM, and (c) 400 mM after 12 h at 80 °C.

The crystal growth is known to depend on the solution concentration, which has influence on both the nucleation rate and diffusion rate. As the concentration of 2-methylimidazole increased from 25 to 50 and 400 mM, the ZIF-8 crystal size changed from the microscale to the nanoscale, indicating a faster nucleation rate at higher concentrations (Figure 5a–c). In addition, the membrane thickness grew from 4 to 11 μm (Figure 5d–f). When the concentration of ligand was 25 mM, it was insufficient to react with all zinc ions in the porous substrate. Consequently, a continuous well-intergrown ZIF-8 membrane was not obtained, α-Al_2_O_3_ particles are exposed on the membrane surface, and the membrane thickness was less than 2 μm (Figure 5a,d). When the ligand concentration increased to 50 mM, a continuous ZIF-8 film with a high surface coverage was obtained (Figure 5b). In this case, the diffusion rate of 2-methylimidazole from 1-octanol solution into aqueous solution was relatively slow. As a result, homogeneous crystal formation was suppressed through the small number of nuclei, while heterogenous nucleation and crystal growth to larger size were favored [41]. Under these conditions, a thinner and well-intergrown ZIF-8 membrane was fabricated (Figure 5b,e). When the concentration of 2-methylimidazole was increased to 400 mM, the diffusion rate of 2-methylimidazole from 1-octanol to aqueous phase significantly increased. A large number of nuclei was formed in the porous surface and diffused through the porous material to react with the Zn ions. The ZIF-8 membrane turned to be thick and grew with nanoscale crystals.

**Figure 6:**
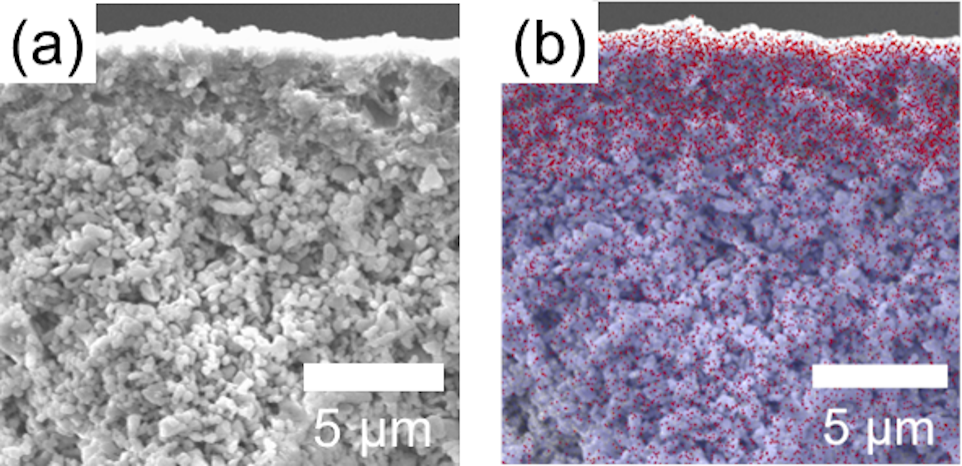
(a) Cross-sectional view FE-SEM image of the synthesized ZIF-8 membrane. (b) Cross-sectional view EDS mapping of the synthesized ZIF-8 membrane (red represents Zn; blue represents Al).

Based on the above results, the optimal ZIF-8 membranes via an interfacial synthesis were achieved with a 2-methylimidazole concentration of 50 mM, reacted at 80 °C for 12 h. The SEM images show that the ZIF-8 crystals were embedded in a dense top layer of the porous α-Al_2_O_3_ disk, suggesting the ZIF-8 particles are small enough to be confined in the substrate, the pore size of which is larger than 100 nm (Figure 6a,b) [42]. These embedded ZIF-8 membranes indicated a greater mechanical strength and thermal stability owing to the intrinsic properties of the porous ceramic support [43].

**Figure 7:**
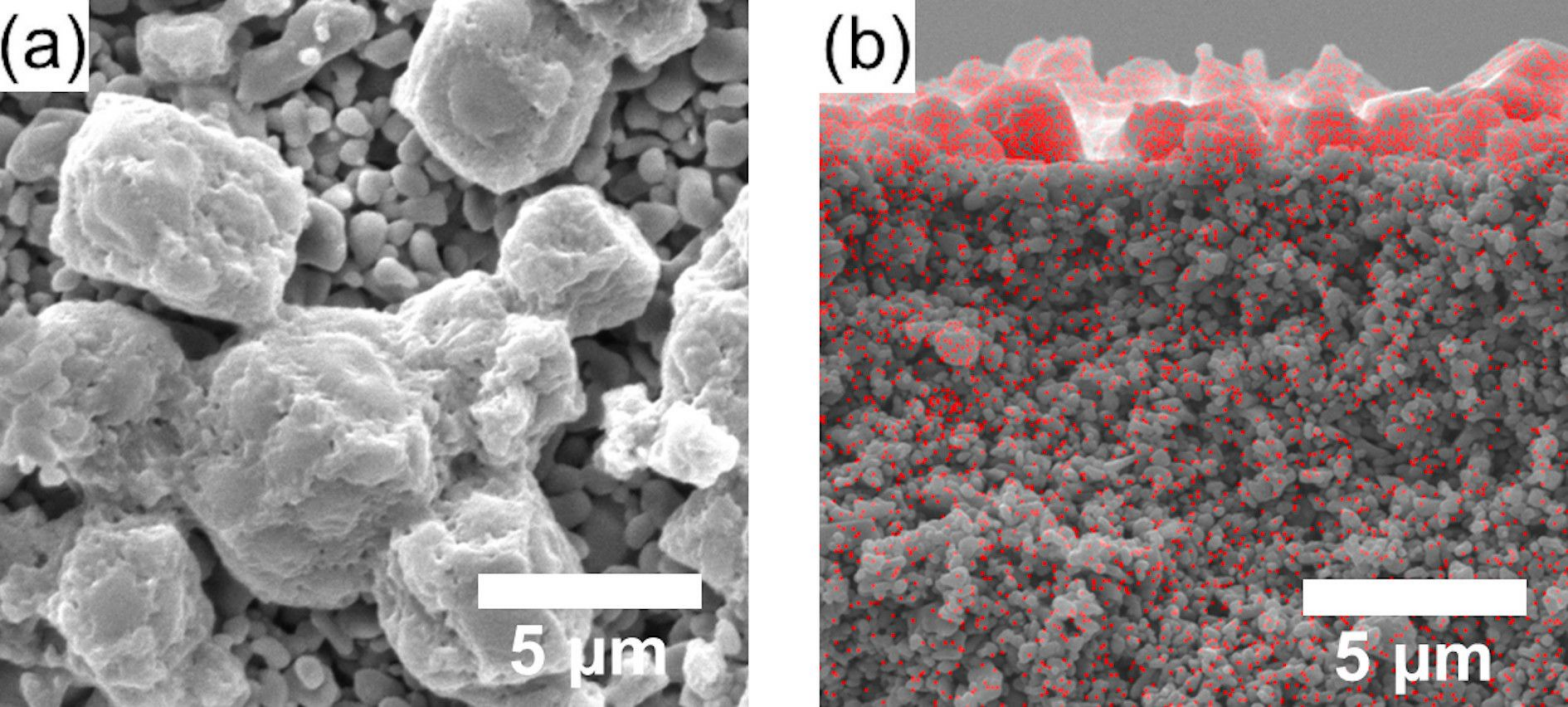
(a) Top-view and (b) cross-sectional SEM images of ZIF-8 membranes fabricated via a counter-diffusion synthesis method (50 mM of 2-methylimidazole at 80 °C for 12 h).

Counter-diffusion synthesis was also adopted in this study to be compared with interfacial synthesis. Defects were observed in the intercrystal gaps on the membrane, and the morphology of the resultant ZIF-8 was different from the one obtained via the interfacial method (Figure 7a) [44]. This is because 1-octanol in the interfacial method facilitated 2-methylimidazole dispersion, while the counter-diffusion synthesis, using water as the solvent, offered less control over ZIF-8 formation. The film was about 1.5 μm thick, on top of the surface of α-Al_2_O_3_ disk rather than embedded into the disk (Figure 7b). Compared to the immiscible solvents in interfacial synthesis, the miscible solvent in counter-diffusion synthesis significantly increased the diffusion rate of the organic linkers to react with Zn ions, which facilitated homogeneous nucleation of ZIF-8 crystals on the surface of α-Al_2_O_3_ disk. Therefore, under the same reaction conditions, more well-intergrown ZIF-8 membranes were obtained via the interfacial synthesis.

### Gas permeation performance of ZIF-8 membranes

Single-gas permeation experiments of CO_2_ and N_2_ were used to evaluate the performance of synthesized ZIF-8 membranes under various conditions. The separation factor increased more than 3.3-fold from 1.28 to 5.49 when the reaction temperature increased from 40 to 80 °C, and the CO_2_ permeance decreased to about a fifth from 2.38 × 10^−7^ to 0.47 × 10^−7^ mol·m^−2^·s^−1^·Pa^−1^ (Table 2, Supporting Information File 1, Figure S2a). These results are in line with the XRD analysis and SEM images, which show that a higher reaction temperature led to higher crystallinity. A further increase of the reaction temperature to 120 °C caused a decrease of the separation factor to 1.77, which was due to the unstable liquid–liquid interface under boiling. In addition, the elevated temperature increased water/1-octanol solubility and resulted in defect formation as revealed by the SEM images.

**Table 2:** Summary of gas (CO_2_/N_2_) separation performance of ZIF-8 membranes from interfacial synthesis (*T* represents reaction temperature; *t* represents reaction time; *c* represents the concentration of the 2-methylimidazole/1-octanol solution).

*T* (°C)	*t* (h)	*c* (mM)	CO_2_ permeance (10^−7^·mol·m^−2^·s^−1^·Pa^−1^)	Separation factor

40	12	50	2.38	1.28
80	12	50	0.47	5.49
120	12	50	1.34	1.77
80	3	50	8.60	1.01
80	6	50	0.59	3.79
80	9	50	0.56	3.97
80	12	50	0.47	5.49
80	15	50	0.41	4.71
80	12	12.5	6.13	1.05
80	12	25	1.99	1.33
80	12	50	0.47	5.49
80	12	100	0.96	2.76
80	12	200	1.67	1.39
80	12	400	2.92	1.26

When the reaction time was increased from 3 to 12 h, the separation factor increased 4.4-fold from 1.01 to 5.49, and the CO_2_ permeance decreased from 8.60 × 10^−7^ to 0.47 × 10^−7^ mol·m^−2^·s^−1^·Pa^−1^ (Table 2, Supporting Information File 1, Figure S2b). The separation factor slightly dropped to 4.71 when the reaction time was prolonged to 15 h. These results can be explained by SEM images and EDS mappings in Figure 4. After 3 h of reaction, the ZIF-8 crystal size was about 0.6 μm and the membrane thickness was around 4 μm. The ZIF-8 crystals continued to grow to a larger grain size of 1.3 μm until equilibrium was reached after 12 h, which caused a denser ZIF-8 layer and thus a higher resistance to CO_2_ permeance.

When the organic linker concentration increased from 12.5 to 400 mM, the CO_2_ permeance first decreased and then increased (Table 2, Supporting Information File 1, Figure S2c). When the linker concentration was less than 25 mM, the number of linkers was insufficient to react with all the Zn ions to form continuous ZIF-8 membranes. At a concentration of 50 mM, the separation factor of the ZIF-8 membrane reached the highest value of 5.49 among all ZIF-8 membranes synthesized with different 2-methylimidazole concentrations. After increasing the concentration from 50 to 400 mM, the separation factor dropped drastically to 1.26, because the diffusion rate of 2-methylimidazole from the organic phase into the aqueous phase outcompeted the ZIF-8 formation rate. As a result, the Zn ions inside the porous substrate were consumed for nanometer-sized ZIF-8 seeds during the initial growing stage, which limited further intergrowth between these seed particles to form continuous ZIF-8 membranes. In addition, thickness of the membranes also increased with increasing 2-methylimidazole concentrations, which reduced CO_2_ permeance. These gas separation performances are well consistent with SEM images and EDS mappings.

Table 3 shows the gas separation performance of ZIF-8 membranes synthesized via interfacial synthesis and counter-diffusion. The two kinds of ZIF-8 membranes were synthesized under the same reaction conditions except for the solvent for 2-methylimidazole. In the case of counter-diffusion, the solvent was deionized water, which was also the solvent for dissolving zinc nitrate hexahydrate. The ZIF-8 membrane synthesized via counter-diffusion showed no separation between CO_2_ and N_2_. Such a low separation factor is ascribed to the defective membranes, which can be seen from the top-view SEM image (Figure 7a). The formation of defects may be associated with faster diffusion rates of 2-methylimidazole in the miscible solvent environment with Zn ions. In contrast, ZIF-8 synthesized via an interfacial method showed a dense and more uniform formation (Figure 5b), which was beneficial for gas separation.

**Table 3:** CO_2_/N_2_ gas separation of ZIF-8 membranes synthesized via two different methods.

Method	CO_2_ permeance (10^−7^·mol·m^−2^·s^−1^·Pa^−1^)	Separation factor

Counter-diffusion synthesis	6.84	0.97
Interfacial synthesis	0.47	5.49

**Figure 8:**
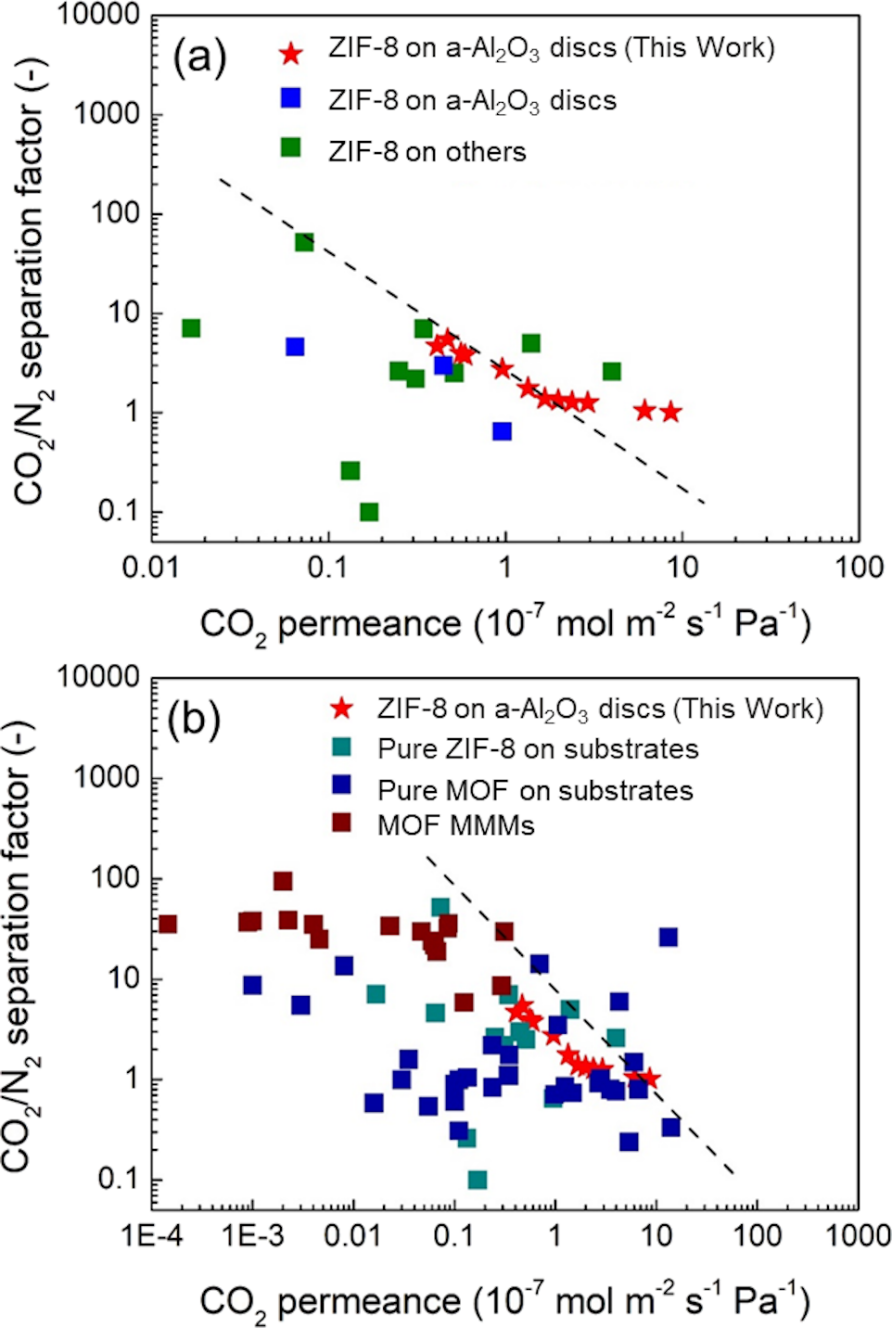
Gas separation performance (CO_2_/N_2_) of the ZIF-8 membranes on α-Al_2_O_3_ disks (red stars, this work) compared with (a) ZIF-8 membranes on α-Al_2_O_3_ disks (blue squares) [46–48] and ZIF-8 membranes on other substrates (green squares) [49–59], and (b) pure ZIF-8 on substrates (turquois squares) [46–59], MOF membranes supported on substrates (blue squares) [50,60–84], and MOF mixed matrix membranes (brown squares) [85–97].

As shown in Figure 8a, the ZIF-8 membranes supported on α-Al_2_O_3_ disks synthesized in this work exhibited comparable CO_2_/N_2_ separation performance with the best ZIF-8 membranes supported on α-Al_2_O_3_ disks reported in literature. These results indicated the reliability and high performance of defect-free ZIF-8 membranes synthesized via the interfacial synthesis method developed in this work. Moreover, the interfacial synthesis method developed in this work was carried out under ambient pressure and prepared in one step. These facile synthesis procedures are favorable for industrial production of MOF membranes.

A few MOF membranes supported on substrates presented a superior CO_2_/N_2_ separation performance over the ZIF-8 membranes synthesized in this work (Figure 8b). The type of MOF has an influence on the gas separation performance, for instance, CAU-1 exhibits a higher CO_2_ adsorption capacity than ZIF-8 [45], which may lead to a higher gas permeance. Compared with MOF mixed-matrix membranes (MMMs), ZIF-8 membranes fabricated in this work showed a lower separation factor but a higher CO_2_ permeance. Dense polymer materials in MMMs tend to lower the gas permeance.

## Conclusion

ZIF-8 membranes embedded in porous α-Al_2_O_3_ disks were successfully fabricated via a simple one-step and low-pressure interfacial synthesis method. It is found that a reaction temperature of 80 °C not only facilitated crystallization of ZIF-8 crystals, but also maintained a stable water/1-octanol interface. The thickness of the membranes changed little after the initial nucleation process, while ZIF-8 crystals further intergrew to form continuous membranes. By mediating the organic linker concentration, defect-free ZIF-8 membranes were obtained. The 2-methylimidazole concentration was then sufficient to react with the Zn ions, but not too high to cause an overly high diffusion rate and the consumption of all Zn ions at the initial nucleation stage. Under the optimized conditions, CO_2_/N_2_ separation factor and CO_2_ gas permeance of the optimized ZIF-8 membrane were 5.49 and 0.47 × 10^−7^ mol·m^−2^·s^−1^·Pa^−1^, respectively. The as-synthesized ZIF-8 membranes exhibited comparable gas separation performance with the best results of the same type of membranes reported in literature. The proposed facile synthesis method in a one-step process at low pressure may favor mass-production in the future.

## Supporting Information

File 1 Additional figures.
