# Supplementary material for: Interfacial nanoarchitectonics for ZIF-8 membranes with enhanced gas separation
Source: Beilstein J Nanotechnol. 2022 Mar 22;13:313–24. doi: 10.3762/bjnano.13.26 (PMC8965340; doi:10.3762/bjnano.13.26)
Supplement: File 1 — Additional figures. [file Beilstein_J_Nanotechnol-13-313-s001.pdf]

BEILSTEIN JOURNAL OF NANOTECHNOLOGY

Supporting Information

for

Interfacial nanoarchitectonics for ZIF-8 membranes with enhanced gas separation

Season S. Chen, Zhen-Jie Yang, Chia-Hao Chang, Hoong-Uei Koh,
Sameerah I. Al-Saeedi, Kuo-Lun Tung and Kevin C.-W. Wu

Beilstein J. Nanotechnol. **2022,** *13,* 313–324. doi:10.3762/bjnano.13.26

Additional figures

License and Terms: This is a supporting information file under the terms of the Creative Commons Attribution License (https://creativecommons.org/licenses/by/4.0). Please note that the reuse, redistribution and reproduction in particular requires that the author(s) and source are credited and that individual graphics may be subject to special legal provisions.
The license is subject to the *Beilstein Journal of Nanotechnology* terms and conditions: (https://www.beilstein-journals.org/bjnano/terms)

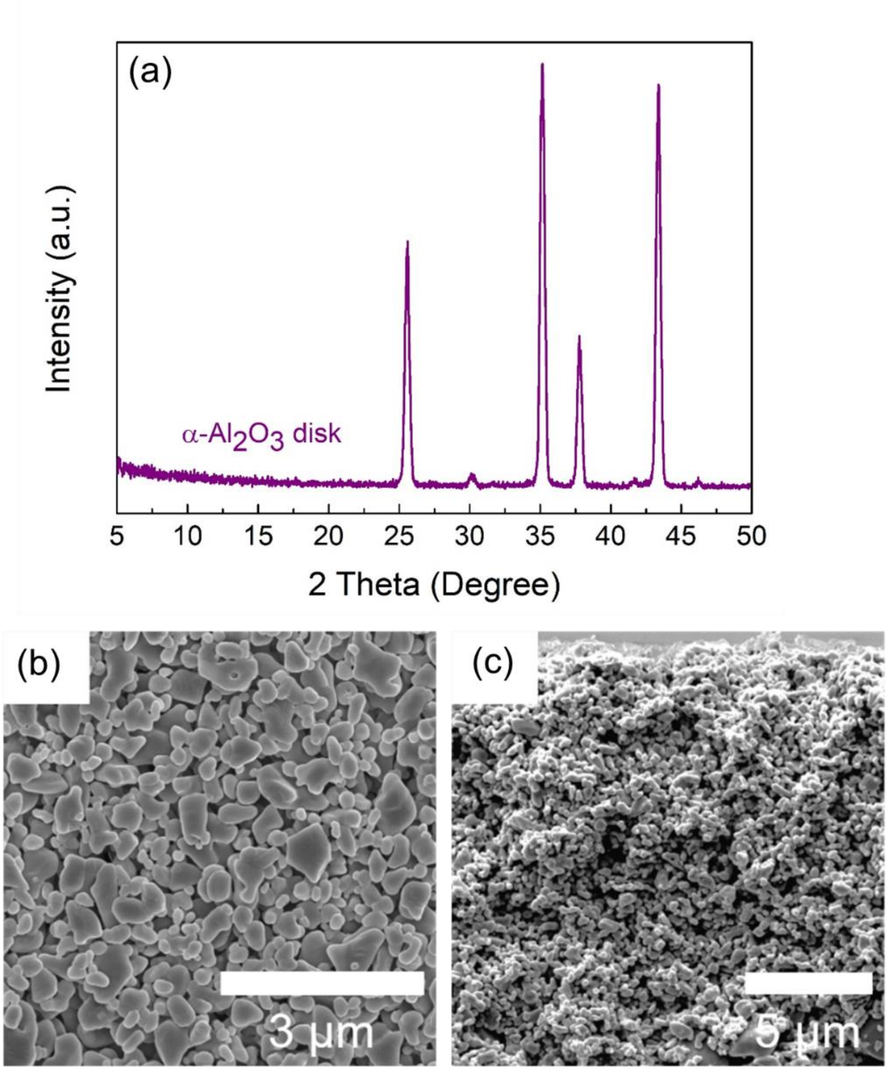

Figure S1: The (a) XRD pattern, (b) top view FE-SEM image, and (c) cross-sectional view FE-SEM image of porous α-Al_2O_3 disk.

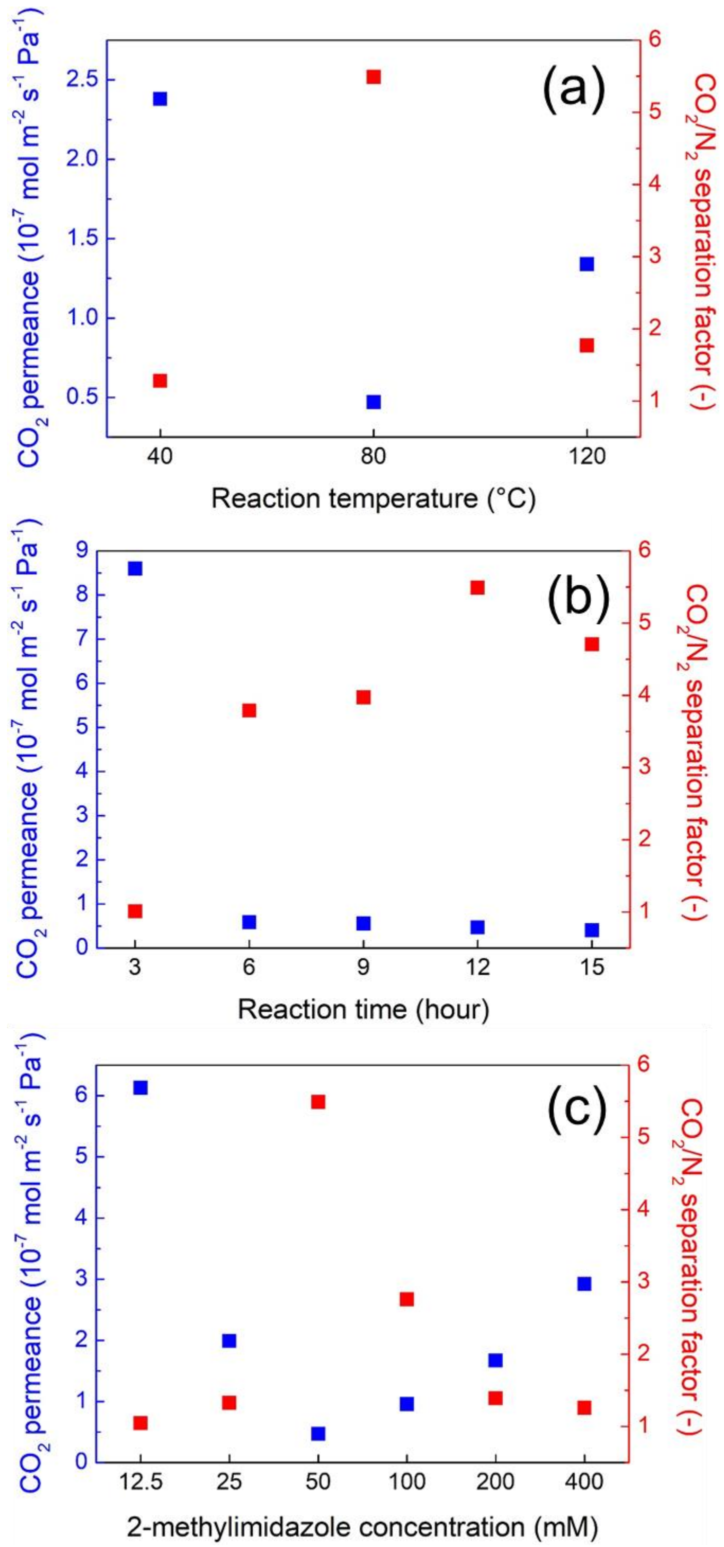

Figure S2: Gas separation (CO_2/N_2) performance of ZIF-8 membranes fabricated at various (a) temperatures, (b) times, and (c) 2-methylimidazole concentrations.
